# Angiotensin-Converting Enzyme Gene D/I Polymorphism in Relation to Endothelial Function and Endothelial-Released Factors in Chinese Women

**DOI:** 10.3389/fphys.2020.00951

**Published:** 2020-09-16

**Authors:** Yuanyuan Lv, Wenying Zhao, Laikang Yu, Ji-Guo Yu, Li Zhao

**Affiliations:** ^1^China Institute of Sport and Health Science, Beijing Sport University, Beijing, China; ^2^Children’s Hospital of Shanxi, Taiyuan, China; ^3^Department of Strength and Conditioning Training, Beijing Sport University, Beijing, China; ^4^Department of Community Medicine and Rehabilitation, Section of Sports Medicine, Umeå University, Umeå, Sweden; ^5^Department of Exercise Physiology, Beijing Sport University, Beijing, China

**Keywords:** *ACE* D/I gene polymorphism, Chinese women, endothelial function, endothelial-released factors, aging, menopause

## Abstract

Many studies have investigated the relationship between angiotensin-converting enzyme (*ACE*) D/I polymorphism and cardiovascular disease or endothelial dysfunction; however, hardly any of these studies has taken aging or menopause into consideration. Furthermore, despite that many studies have examined the regulatory effects of endothelial-released factors (ERFs) on endothelial function, no study has evaluated the relationship between ERFs and endothelial function with respect to *ACE* D/I polymorphism and menopause status. To answer these questions, 391 healthy Chinese women over a wide range of ages (22–75 years) were enrolled and divided into pre-menopause group and post-menopause group. After *ACE* D/I genotype being identified, the women were then classified into either DI/II or DD genotype. Flow-mediated dilatation (FMD) of brachial endothelium and plasma levels of ERFs: nitric oxide (NO), endothelin-1 (ET-1), and angiotensin II (Ang II) were measured. The results showed that frequencies of *ACE* D/I genotypes were in accordance with the Hardy-Weinberg equilibrium, and the frequency of I allele was higher than D allele. In pre-menopause group, FMD was significantly higher in women of DI/II than DD (*P* = 0.032), and age-dependent in both genotypes (DD, *P* = 0.0472; DI/II, *P* < 0.0001). In post-menopause group, FMD was similar between women of DI/II and DD, and age-dependent only in women of DI/II (*P* < 0.0001). In pre-menopause group, Ang II level was significantly higher in women of DD than DI/II (*P* = 0.029), and FMD was significantly correlated with all ERFs in women of DD (NO, *P* = 0.032; ET-1, *P* = 0.017; Ang II, *P* = 0.002), but only with Ang II in women of DI/II (*P* = 0.026). In post-menopause group, no significant difference was observed in any ERF between women of DI/II and DD, and FMD was only significantly correlated with ET-1 in women of DD (*P* = 0.010). In summary, FMD in women of DI/II was superior to DD in pre-menopause and more age-dependent than DD in post-menopause, and FMD was closely associated with ERFs. In conclusion, Chinese women of DI/II seem to have lower risk than DD in pre-menopause, but similar risk as DD in post-menopause in developing cardiovascular disease.

## Introduction

Endothelium-dependent vasodilation impairment is believed to be the initial factor in the pathogenesis of atherosclerosis, according to the “endothelial damage response theory” (Gimbrone and García-Cardeña, 2016; Ungvari et al., 2018b), and reduction in endothelial function presents a strong correlation with aging, especially after the age of 45 years (Shi et al., 2014; Sepúlveda et al., 2017; Jia et al., 2019) when menopausal transition generally occurs. Thus, endothelial dysfunction represents one of the earliest signs of developing cardiovascular and cerebrovascular diseases, such as atherosclerosis, coronary heart disease, hypertension (Rossman et al., 2018; Ungvari et al., 2018b). Flow-mediated dilation (FMD) of the brachial artery is currently a widely used parameter in evaluating vasodilation (Aeschlimann et al., 2004; Thijssen et al., 2011) and in clinical studies to independently predict the risk of cardiovascular diseases (Takase et al., 1998; Corretti et al., 2002; Inaba et al., 2010; Green et al., 2011; Donato et al., 2018; Nemoto et al., 2019).

Angiotensin-converting enzyme (*ACE*) is a single-chain polypeptide acid glycoprotein and was first discovered in the vascular endothelium, where it is often in a membrane-bound form (Kim et al., 2001). *ACE* is a key enzyme of renin-angiotensin system, which together with kallikrein-kinin system is important in maintaining physiological functions of the heart, blood vessels, and kidneys through the regulation of blood pressure, blood flow, homeostasis, and the vasomotor system (Bader, 2010; Masuyer et al., 2012). In the 16th intron of the *ACE* gene, there is a 287 bp Alu repeat insertion/deletion, and when the *ACE* allele contains this fragment, it is called “I” (insertion type). If not, it is called “D” (deletion type); thus, *ACE* has three different genotypes: type II, DI, and DD (Kim et al., 2001).

Angiotensin-converting enzyme gene D/I polymorphism has been shown to present significant linkage disequilibrium, and DD genotype has the highest *ACE* activity, DI genotype of moderate, and II genotype of the lowest (Cambien et al., 1988; Rigat et al., 1990). Thus, high number of D allele indicates high *ACE* activity and vice versa (Masuyer et al., 2012). Although *ACE* activity varies greatly among individuals, it remains generally constant in different tissues/organs of the same individual as it is hardly affected by external factors (Shafiee et al., 2010). Recent studies have revealed some differences in distribution of *ACE* D/I gene polymorphism between different ethnic groups and even within the same ethnic group (Alhenc-Gelas et al., 1991; Cambien and Evans, 1995).

Angiotensin-converting enzyme gene D/I polymorphism has been shown to be associated with various cardiovascular diseases (Sayed-Tabatabaei et al., 2006). In a previous study, the frequency of *ACE* D allele in myocardial infarction subjects has been shown to be significantly higher than in healthy control subjects (Cambien et al., 1992). The authors suggested that the *ACE* D allele was an independent risk factor for myocardial infarction in European. The *ACE* DD genotype has also been reported to be highly correlated with the incidence of hypertension (Kato et al., 2011) and cerebrovascular disease/stroke (Kalita et al., 2011). In addition, *ACE* gene polymorphism has been shown to be closely associated with endothelial dysfunction (Tanriverdi et al., 2007). In a study of post-menopausal women, endothelial function was observed to be significantly lower in *ACE* DD than in *ACE* II (Méthot et al., 2006). Similarly, in another study of male patients with chronic obstructive pulmonary disease, a significantly lower incidence of endothelial dysfunction (FMD < 10%) was observed in patients of *ACE* DI/II than in *ACE* DD (Kuzubova et al., 2013). It is well-known that endothelial function is age-dependent (Ungvari et al., 2018a), and estrogen has a strong protective effect on endothelial function, which decreases significantly after menopause (Taddei et al., 1996). However, to our knowledge no study to date has investigated the relationship between *ACE* polymorphism and endothelial function in women following aging, particularly across the age of menopause.

Physiologically, the vascular endothelium can produce and release a variety of vasoactive substances, such as nitric oxide (NO), angiotensin II (Ang II), endothelin-1 (ET-1) (Donato et al., 2018; Stanhewicz et al., 2018). These substances can cause vasodilation or contraction of vascular smooth muscles, thus regulating vascular tone (Godo et al., 2016; Somani et al., 2019). NO is the most important factor in the vascular endothelial vasodilation system and plays a fundamental role in regulating vascular resistance and tissue perfusion. ET-1 is the strongest vasoconstrictor produced by the vascular endothelium (D’Orléans-Juste et al., 2008) and plays an important role in vascular dysfunction during aging. Ang II can increase total peripheral resistance and lead to high blood pressure, thus playing a major role in the occurrence of cardiovascular disease (Al-Hazzani et al., 2014). Overall, NO, ET-1, and Ang II are closely related and interact with each other to regulate the normal function of vascular endothelium (Godo et al., 2016; Somani et al., 2019).

Despite the fact that many studies have examined the regulatory effects of endothelial-released factors (ERFs) on endothelial function, no study has ever examined the differences of these factors in affecting endothelial function in relation to *ACE* gene D/I polymorphism, especially in women across the life stage of menopause. To answer these questions, the present study examined the distribution of *ACE* gene D/I genotypes in a large group of Chinese women including both pre-menopause stage and post-menopause stage. Endothelial-dependent vasodilation function and ERFs were examined. We aimed to reveal the relationship between the *ACE* gene D/I genotype, endothelial function, and plasma levels of ERFs following aging in pre- and post-menopausal women. We hope the results will help us to know more about the potential risk in developing cardiovascular disease in Chinese women of different *ACE* gene D/I genotypes at different menopause stages.

## Materials and Methods

### Subjects

A total of 391 healthy women of Han nationality (22–75 years) from the local region (Beijing, China) were recruited for this cross-sectional study. Questionnaires with questions about health status, medicines intake, menstrual status, and use of hormonal contraceptives were distributed to all participants. Women with diabetes, cardiovascular diseases, or other metabolic diseases, or taking medicines for blood pressure control or blood lipid regulation were excluded. We used the World Health Organization’s definition of menopause, i.e., the cessation of menstruation being longer than 12 months. On basis of menopause status of each individual, the subjects were divided into two groups: pre-menopause group (*n* = 155) and post-menopause group (*n* = 236).

### Ethics Statement

The protocol used in this study was approved by the Ethics Committee of Beijing Sport University (2019014H). The study was in accordance with the recommendations of the Declaration of Helsinki. All participants were informed about the study and written informed consent was obtained from all the participants.

### *ACE* Gene D/I Polymorphism Identification and Classification

Detailed description of the procedure of *ACE* gene D/I genotype identification has been described previously (Comey et al., 1994; Sun et al., 2018; He et al., 2019). DNA samples were extracted from buccal mucosal cells by cotton swabs. *ACE* gene D/I polymorphism was determined using polymerase chain reaction (PCR). DNA samples were extracted using 5% chelex-100 (165 μl) and proteinase K (3 μl, 20 mg/μl) and amplified using the forward primer of 5’-CTG GAG ACC ACT CCC ATC CTT TCT-3’ and the reverse primer 5’-GAT GTG GCC ATC ACA TTC GTC AGA T-3’ (Lian et al., 2012). The PCR reaction system (15 μl) consisted of ddH_2_O (10.8 μl), dNTP (1.2 μl), 10 × buffer (1.5 μl), TAKARA HS Taq polymerase (0.1 μl), each primer (0.2 μl), and template (1.0 μl). The DNA was amplified by 35 cycles using PCR Thermal (iCycler, Bio-Rad, United States). Every cycle was started with pre-denaturation at 94°C for 5 min, followed by 30 s of denaturation at 94, 55, and 72°C. The amplification was ended with a final elongation of 10 min at 72°C and kept at 15°C. To identify the *ACE* D/I genotype, 2 μl PCR amplicon was electrophoresed using a 2.5% agarose gel with the presence of a 190 bp fragment for D allele and a 470 bp fragment for I allele.

### Measurement of Brachial Endothelium-Dependent Vasodilation – FMD

Details of the procedures of FMD measurement have been described previously (Celermajer et al., 1992, 1994; Yeboah et al., 2009; Larsen et al., 2016). Briefly, the subjects rested supine for at least 10 min, and the forearm was pressurized below the elbow joint at 280–300 mmHg for 5 min with an inflatable tourniquet and then suddenly deflated. Two-dimensional images of the brachial artery were obtained at baseline (D_0_) and between 45 to 60 s after cuff release (D_1_) with a 7.5 to 13 MHz high frequency linear array probe (GE Vivid 7, United States; D’Andrea et al., 2007). An electrocardiogram was recorded synchronously using an electrocardiograph (Custo med cardio 100, Germany). All measurements were performed at the end of the diastole to reduce the effect of possible vascular compliance. The R-wave of the ECG was used as the standard of the end-diastole. FMD was calculated using the formula: FMD = (D_1_–D_0_)/D_0_ × l00%. Conventionally, FMD could be categorized into three different levels, FMD > 10%, 7% ≤ FMD ≤ 10%, FMD < 7%, representing normal, mildly abnormal, and moderately- to severely abnormal vascular endothelial function, respectively (Neunteufl et al., 1997; Berzigotti et al., 2013; Basyigit et al., 2015).

### Measurements of ERFs

Blood samples were collected from each subject from the antecubital vein in the morning after overnight fasting. For Ang II analysis, blood sample of 5 ml was collected in Vacutainer tube containing ethylene diamine tetraacetic acid (EDTA) for anticoagulation, and then three different types of enzyme inhibitors (EDTANa, 0.3 M, 250 μl; 8-hydroxyquinoline, 0.34 M, 50 μl; dimercaptopropanol, 0.32 M, 25 μl) were added into the tube. Another blood sample of 3 ml was collected for analysis of ET-1 and NO. NO was measured using the nitric acid reductase method (Fabricio et al., 2017). Ang II (Huang et al., 2004b) and ET-1 (Al-Fakhri et al., 2003) were measured using radioimmunoassay. The analysis kits for ET-1 and NO were provided by the Nanjing Jincheng Institute of Biological Engineering (Nanjing, China), and the kit for Ang II was provided by the North Institute of Biotechnology (Beijing, China). The analysis procedures have been described in previous study (Ramzy et al., 2006a,b; González-González et al., 2018). NO, Ang II and ET-1 were analyzed at the cardiovascular laboratory, Beijing Sport University, following standardized procedures.

### Statistical Analysis

All statistical analyses were performed using SPSS (version 20.0, International Business Machines Corporation, United States). Continuous variables were shown as mean ± standard deviation, and enumeration data were shown as rates. Chi-Square (χ^2^) tests were used in Hardy-Weinberg equilibrium calculations of the *ACE* D/I allele frequencies of the subjects and in comparisons of the proportions of women at different FMD levels between groups (When the sample size was less than five, Yates’ correction was applied). Comparisons of continuous variables between the pre-menopause group and the post-menopause group or between the *ACE* DI/II and the *ACE* DD genotypes were performed using independent-samples *t*-test. Multivariate analysis of variance was used to evaluate the determinants of *ACE* gene D/I polymorphism, age, and menopause status on FMD. Multivariate regression analysis was used to evaluate the determinants of ERFs on FMD. A two-tailed *P*-value of less than 0.05 was considered statistically significant.

## Results

### Anthropometric Data and *ACE* Genotype Classification

Based on their menopause stages, the women were classified into either the pre-menopause group or the post-menopause group (Table 1). The frequencies of women of both D allele and I allele were calculated for each group, and a χ^2^ test showed that the distributions were in accordance with the Hardy-Weinberg equilibrium. General anthropometry and blood pressure are shown in Table 2. Comparisons of the data between women of *ACE* DI/II and *ACE* DD within each group showed that in pre-menopause group, the average age of women with *ACE* DI/II was significantly higher than that of *ACE* DD (*P* = 0.000), and both systolic blood pressure (SBP) and diastolic blood pressure (DBP) were significantly lower in women of DI/II than in women of *ACE* DD (*P* = 0.009 and *P* = 0.015, respectively). Comparisons of the data between pre-menopause group and post-menopause group of the same *ACE* genotype revealed that women of *ACE* DI/II had significantly higher of both SBP and DBP in post-menopause group than in pre-menopause group (*P* = 0.012 and *P* = 0.006, respectively).

**TABLE 1:** Distribution of *ACE* gene polymorphism of pre- and post-menopause groups and the Hardy-Weinberg equilibrium.

**Groups**	**Proportions of phenotype**			**Proportions of D/I allele**		**χ^2^ (*p*)**
	**DD**	**DI**	**II**	**D**	**I**	
Pre-menopause (*n* = 155)	10.32% (*n* = 16)	47.74% (*n* = 74)	41.94% (*n* = 65)	34.19% (*n* = 106)	65.81% (*n* = 204)	0.259 (0.879)
Post-menopause (*n* = 236)	12.71% (*n* = 30)	46.19% (*n* = 109)	41.10% (*n* = 97)	35.81% (*n* = 169)	64.19% (*n* = 303)	0.003 (0.999)
Pre vs. post χ^2^ (*p*)	0.514 (0.473)	0.091 (0.763)	0.027 (0.870)	0.115 (0.735)	0.115 (0.735)	

*A chi-square (χ^2^) test was used for genotype distribution. DD, homozygous deletion; DI, DI heterozygote; II, homozygous insertion; D/I, deletion/insertion; χ^2^, chi-square statistic. Pre vs. Post, comparison of pre- and post-menopausal women.*

**TABLE 2:** Comparisons of anthropometric parameters between angiotensin-converting enzyme insertion/deletion subgroups of the pre- and post-menopause groups (M ± SD).

**Characteristics**	**Pre-menopause (*n* = 155)**			**Post-menopause (*n* = 236)**			**Pre vs. post (DD)**	**Pre vs. post (DI/II)**
	**DD (*n* = 16)**	**DI/II (*n* = 139)**	***P***	**DD (*n* = 30)**	**DI/II (*n* = 206)**	***P***	***P***	***P***
Age (y)	42.88 ± 1.93	46.42 ± 3.61	0.000^a^	60.77 ± 6.61	61.88 ± 6.15	0.360		
Height (cm)	160.81 ± 7.12	157.94 ± 6.18	0.053	158.02 ± 5.66	157.03 ± 6.06	0.390	0.293	0.801
Weight (Kg)	61.97 ± 7.98	61.67 ± 7.13	0.765	61.09 ± 7.50	59.74 ± 7.78	0.373	0.658	0.216
SBP (mm Hg)	132.09 ± 9.85	118.95 ± 9.02	0.009^a^	131.10 ± 10.91	129.68 ± 9.85	0.899	0.117	0.012^d^
DBP (mm Hg)	76.05 ± 7.86	68.37 ± 6.15	0.015^b^	77.43 ± 8.51	75.69 ± 9.38	0.436	0.300	0.006^c^

*Independent-samples *t-*tests were used to compare continuous variables between pre- and post-menopause groups, and between *ACE* DI/II and *ACE* DD genotypes. SBP, systolic blood pressure; DBP, diastolic blood pressure; DD, homozygous deletion; DI/II, I allele carriers. Pre vs. Post (DD), comparison of pre- and post-menopausal women in DD group; Pre vs. Post (DI/II), comparison of pre- and post-menopausal women in I allele carriers. ^*a*^Significantly different between DD and II/ID groups, *P* < 0.01. ^*b*^Significantly different between DD and II/ID groups, *P* < 0.05. ^*c*^Significantly different between pre- and post-menopause groups, *P* < 0.01. ^*d*^Significantly different between pre- and post-menopause groups, *P* < 0.05.*

### Results and Comparisons of FMD Measurements

The results of FMD are shown in Table 3. Comparisons in mean values of FMD between women of *ACE* DD and *ACE* DI/II within pre-menopause group and post-menopause group showed significant difference only in pre-menopause group, where FMD mean value was significantly lower in women of *ACE* DD than *ACE* DI/II (*P* = 0.032). Comparisons in mean values of FMD in women of the same *ACE* genotype between pre-menopause group and post-menopause group revealed significant difference only in women of *ACE* DI/II, which presented significantly higher FMD mean value in pre-menopause group than in post-menopause group (*P* = 0.000).

**TABLE 3:** Results and comparisons of FMD measurements.

**FMD (%)**	**Pre-menopause (*n* = 155)**			**Post-menopause (*n* = 236)**			**Pre vs. Post (DD)**	**Pre vs. Post (DI/II)**
	**DD (*n* = 16)**	**DI/II (*n* = 139)**	***P***	**DD (*n* = 30)**	**DI/II (*n* = 206)**	***P***	***P***	***P***
M ± SD	8.89 ± 2.56	10.20 ± 2.27	0.032^b^	8.62 ± 1.62	8.43 ± 2.91	0.365	0.172	0.000^c^
>10	43.8% (7)	54.7% (76)	0.407	6.7% (2)	21.4% (44)	0.099	0.009^c^	0.000^c^
≤10, ≥7	31.3% (5)	41.0% (57)	0.451	60.0% (18)	35.4% (73)	0.010^b^	0.063	0.295
<7	25% (4)	4.3% (6)	0.008^a^	33.3% (10)	43.2% (89)	0.306	0.804	0.000^c^

*Independent-samples *t*-tests were used to compare FMD mean values between pre- and post-menopause groups and between *ACE* DI/II and *ACE* DD genotypes. Chi-Square (χ2) tests were used to compare the proportions of women at different FMD levels between pre- and post-menopause groups, and between ACE DI/II and ACE DD genotypes (When sample size was less than five, Yates’ correction was applied). FMD, flow-mediated dilatation. FMD > 10% is normal; 7% ≤ FMD ≤ 10% is mild abnormality; FMD < 7% is moderate to severe abnormality. Pre vs. Post (DD), comparison of pre- and post-menopausal women in DD group; Pre vs. Post (DI/II), comparison of pre- and post-menopausal women in I allele carriers. ^*a*^Significantly different between DD and DI/II groups, *P* < 0.01. ^*b*^Significantly different between DD and DI/II groups, *P* < 0.05. ^*c*^Significantly different between pre-and post-menopausal women, *P* < 0.01.*

Frequencies of women of both *ACE* genotypes in both groups at different FMD levels (FMD < 7%; 7% ≤ FMD ≤ 10%; FMD > 10%) are shown in Table 3. In pre-menopause group, the highest proportions of women for both *ACE* genotypes were at normal FMD level (FMD > 10%; DD, 43.8% and DI/II, 54.7%), and the proportion of women at moderately- to severely abnormal FMD level (FMD < 7%) was significantly higher in *ACE* DD than in *ACE* DI/II (*P* = 0.008). In post-menopause group, the highest proportion of women of *ACE* DD (60%) was at mildly abnormal FMD level (7% < FMD < 10%), whereas the highest proportion of women of *ACE* DI/II (43.2%) was at moderately- to severely abnormal FMD level (FMD < 7%).

Comparisons of proportions of women of the same *ACE* genotype at different FMD levels between pre-menopause group and post-menopause group showed that at normal FMD level (FMD > 10%), the proportions of women for both *ACE* genotypes in post-menopause group were significantly lower than that of respective *ACE* genotype in pre-menopause group (DD, *P* = 0.009 and DI/II, *P* = 0.000; Table 3). By contrast, women at mildly abnormal FMD level (7% ≤ FMD ≤ 10%), no significant difference was observed in women of either *ACE* DD or *ACE* DI/II between pre-menopause group and post-menopause group (DD, *P* = 0.063 and DI/II, *P* = 0.295). At moderately- to severely abnormal FMD level (FMD < 7%), significant difference was observed only in women of *ACE* DI/II, who presented significantly higher proportion in post-menopause group than in pre-menopause group (*P* = 0.000).

### Multivariate Analysis of Variance of FMD With Independent Variables of *ACE* Genotype, Age, and Menopause Status

Results of multivariate analyses of variance are shown in Table 4. Among the three independent variables, only age and menopause status were significantly correlated with FMD (*P* = 0.006 and *P* = 0.012, respectively). In addition, interactions of *ACE* genotype with both age and menopause status presented significant correlations with FMD (*P* = 0.012 and *P* = 0.015, respectively).

**TABLE 4:** Multivariate analysis of variance of FMD with independent variables of *ACE* genotype, age, and menopause status.

**Variable**	**F-statistic**	***P* value**
Age	5.354	0.006
*ACE* genotype	3.093	0.081
Age*ACE genotype	6.070	0.012
Menopause status	4.962	0.026
Menopause status *ACE genotype	8.077	0.015

Multivariate analysis of variance was used to evaluate the effects of *ACE* gene D/I polymorphism, age, and menopause status on FMD. *ACE*, angiotensin-converting enzyme; Age^∗^*ACE* genotype, interaction of Age and ACE genotype; Menopause status *ACE genotype, interaction of menopause status and ACE genotype.

### Correlation Analysis Between FMD and Age in Women of the Pre-menopause Group and the Post-menopause Group

**FIGURE 1:**
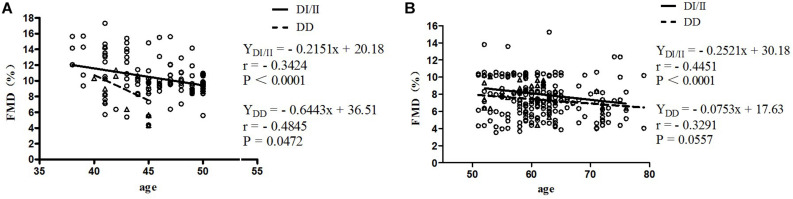
Correlation analysis between FMD and age in women of the pre-menopause group **(A)** and the post-menopause group **(B)**. FMD, flow-mediated dilatation; DD, homozygous deletion; DI/II, I allele carriers. The subjects were classified as belonging to pre- or post-menopause groups according to their menopause status. Age as the abscissa and FMD as the ordinate, two-dimensional FMD plot of women with different *ACE* genotypes in different menopause stages were made; linear regression was performed for FMD and age. **(A)** Pre-menopause group; **(B)** Post-menopause group.

Results of correlation analyses between FMD and age for women of *ACE* DI/II and *ACE* DD in pre-menopause group and post-menopause group are shown in Figure 1. In pre-menopause group, FMD was low, yet, significantly and negatively correlated with age for women of both *ACE* genotypes; (DD, *r* = −0.4845, *P* = 0.0472; DI/II, *r* = −0.3424, *P* < 0.0001). In post-menopause group, FMD was also low and negatively correlated with age for women of both *ACE* genotypes (DD: *r* = −0.3291; DI/II: *r* = −0.4451), but significant correlation was only observed in women of DI/II (*P* < 0.0001).

### ERFs Levels and Comparisons

In pre-menopause group, among the three ERFs, only Ang II level presented significant difference between women of *ACE* DI/II and *ACE* DD, i.e., significantly higher in women of *ACE* DD than in *ACE* DI/II (*P* = 0.029; Table 5). In post-menopause group, none of the three ERFs presented significant difference between women of *ACE* DI/II and *ACE* DD. Further comparisons of ERFs levels in women of the same *ACE* genotype between pre- and post-menopause groups revealed no significant difference in any factor in women of *ACE* DD, whereas in women of *ACE* DI/II, only ET-1 level presented significant difference, i.e., significantly higher in post-menopause group than in pre-menopause group (*P* = 0.014).

**TABLE 5:** ERFs levels and comparisons (M ± SD).

**Genotype**	**Pre-menopause (*n* = 155)**			**Post-menopause (*n* = 236)**			**Pre vs. Post (DD)**	**Pre vs. Post (DI/II)**
	**DD (*n* = 16)**	**DI/II (*n* = 139)**	***P***	**DD (*n* = 30)**	**DI/II (*n* = 206)**	***P***	***P***	***P***
NO (μmol/l)	61.239.78	66.1910.04	0.087	64.298.40	64.328.21	0.986	0.272	0.107
ET-1 (ng/l)	70.126.00	66.007.84	0.337	74.529.85	70.4610.21	0.202	0.379	0.014^b^
Ang II (pg/ml)	135.5114.29	122.0712.89	0.029^a^	126.8918.35	124.0017.99	0.540	0.202	0.463

*Independent-samples *t*-tests were used to compare continuous variables between pre- and post-menopause groups, and between *ACE* DI/II and *ACE* DD genotypes. DD, homozygous deletion; DI/II, I allele carriers. NO, nitric oxide; ET-1, endothelin-1; Ang II, angiotensin II. Pre vs. Post (DD), comparison of pre- and post-menopausal women in DD group; Pre vs. Post (DI/II), comparison of pre- and post-menopausal women in I allele carriers. ^*a*^Significantly different between DD and DI/II groups, *P* < 0.05. ^*b*^Significantly different between pre- and post-menopause groups, *P* < 0.05.*

### Multivariate Regression Analysis Between FMD and ERFs

We used FMD as a dependent variable, and ERFs as independent variables for multivariate regression analyses, the results are shown in Table 6. In pre-menopause group, Ang II had significant correlation with FMD in women of both *ACE* genotypes (DD, *P* = 0.002; DI/II, *P* = 0.026), whereas NO and ET-1 had significant correlations with FMD only in women of *ACE* DD (*P* = 0.032 and *P* = 0.017, respectively). In post-menopause group, only ET-1 presented significant correlation with FMD in women of *ACE* DD (*P* = 0.010).

**TABLE 6:** Multivariate regression analysis between FMD and ERFs.

**Variable**	**Pre-menopause**		**Post-menopause**	
	**DD (*P*-value)**	**DI/II (*P-*value)**	**DD (*P*-value)**	**DI/II (*P*-value)**
NO	0.032	0.301	0.614	0.442
ET-1	0.017	0.439	0.010	0.308
Ang II	0.002	0.026	0.759	0.647

*Multivariate regression analysis was used to evaluate the effects of ERFs on FMD. DD, homozygous deletion; DI/II, I allele carriers. NO, nitric oxide; ET-1, endothelin-1; Ang II, angiotensin II.*

## Discussion

### *ACE* D/I Polymorphism in Relation to FMD, Aging, and Menopause Status

The study revealed that *ACE* D/I allele frequencies in Chinese women of Han nationality were in accordance with the Hardy-Weinberg equilibrium (see Table 1). The frequency of *ACE* I allele was higher than that of *ACE* D allele, similar to previous studies on the same ethnic population (Gao et al., 2014; Cai et al., 2019). However, this was different from other nations in the world, where the frequency of *ACE* D allele was in general higher than that of *ACE* I allele (Moran et al., 2005; Daniela et al., 2008; Dankova et al., 2009). Nevertheless, it has been long recognized that the frequencies of *ACE* D/I allele were ethnic and regional (Wiwanitkit, 2004; Saab et al., 2007).

Comparisons of FMD mean values between different *ACE* genotypes within pre-menopause group and post-menopause group revealed that in pre-menopause group, FMD mean value was significantly higher in women of *ACE* DI/II than in *ACE* DD, but in post-menopause group, no significant difference was observed (see Table 3). In addition, comparisons of FMD mean values in women of the same *ACE* genotype between pre-menopause group and post-menopause group revealed that FMD in women of *ACE* DI/II was significantly higher in pre-menopause group than in post-menopause group, but FMD in women of *ACE* DD did not present significant difference. The results indicate clearly that in pre-menopause group, women of *ACE* DI/II had better endothelial function of FMD than those of *ACE* DD, but that advantage disappeared in post-menopause stage. In support of this, in the pre-menopause group, women of *ACE* DI/II had significantly lower values of both systolic- and diastolic blood pressure than those of *ACE* DD, whereas in the post-menopause group, women of the two *ACE* genotypes did not present significant difference in either systolic- or diastolic blood pressure (see Table 2). The conclusion was also supported by the comparisons at different FMD levels (see Table 3). In pre-menopause group, percentage of women at moderately- to severely abnormal FMD level (FMD < 7%) was significantly lower in women of *ACE* DI/II than in women of *ACE* DD, whereas in post-menopause group, no significant difference was observed. Interestingly, in pre-menopause group, women of *ACE* DI/II were on average older than that of *ACE* DD (see Table 2), strengthening further our conclusion that women of *ACE* DI/II had advantage in FMD over women of *ACE* DD.

Previous studies have shown that *ACE* DD had close association with a high incidence of cardiovascular disease or endothelial dysfunction (Alvarez et al., 2001; Brand and van der Schouw, 2010; Kuzubova et al., 2013; Al-Hazzani et al., 2014; Amara et al., 2018). In the present study, only the result of pre-menopause group was consistent with previous studies, i.e., women of *ACE* DD had lower FMD than women of *ACE* DI/II, and in post-menopause group, women of the two *ACE* genotypes did not present significant difference in FMD. Interestingly, in another study on post-menopausal women, endothelial function was significantly lower in *ACE* DD than in *ACE* II (Méthot et al., 2006). It has been shown that different ethnic groups may vary in age of menopause (Santoro and Chervenak, 2004), whereas menopause is closely associated with different hormones and factors such as ERFs (Kang et al., 2011), exerting different regulatory effects on FMD. Thus, it would be interesting to compare the difference in endothelial function in relation to menopause status, aging, and *ACE* D/I genotype between different ethnic groups.

Previous studies have shown a high correlation between FMD, menopause status, and aging (Ungvari et al., 2018b). In support of this, the present study observed that in post-menopause group, percentages of women of both *ACE* genotypes at normal FMD level (FMD > 10%) were significantly lower than those of respective *ACE* genotypes in pre-menopause group (see Table 3). In fact, when all of the women were taken as one group, no significant correlation between FMD and *ACE* genotype was observed (see Table 4). However, interactions of *ACE* genotype with both age and menopause status presented significant correlations with FMD (see Table 4). As age and menopause are intertwined, it is no surprise that if age shows a statistical association, then menopause will too. To break down the intertwining, we performed further correlation analyses between FMD and age for women of both *ACE* genotypes in both pre- and post-menopause groups. The results revealed that in pre-menopause group, FMD was significantly correlated with age in women of both *ACE* genotypes, but in post-menopause group, significant correlation between FMD and age was only observed in women of *ACE* DI/II (see Figure 1). A simple comparison of the slopes of the regressions in women of the same *ACE* D/I genotype between pre- and post-menopause groups revealed that the slopes in women of *ACE* DI/II were similar (pre-menopause group: −0.2151 vs. post-menopause groups: −0.2521), indicating that FMD in women of *ACE* DI/II declined at similar speed following aging in pre- and post-menopause stages. However, the slopes for women of *ACE* DD were much deeper in pre-menopause group (−0.6443) than in post-menopause group (−0.0753), suggesting that FMD in women of *ACE* DD declined much faster following aging in pre-menopause stage than in post-menopause stage. To our knowledge, this is the first study to date to investigate the relationship between FMD and aging in women of different menopause stages and different *ACE* D/I genotypes. Our results strongly suggest the importance of menopause status in affecting FMD following aging in women of different *ACE* D/I genotypes.

Flow-mediated dilatation has been shown to be significantly lower in post-menopausal women than in pre-menopausal women (Moreau et al., 2012, 2013; Santos-Parker et al., 2017). The reduction in vascular function in the transition to menopause has been suggested to be due to decline of gonadal hormones (Moreau, 2018), especially estrogen (Witkowski and Serviente, 2018). However, the hypothesis could not fully explain our observations, because in post-menopause group, significantly lower FMD mean value was observed only in women of *ACE* DI/II, not in women of *ACE* DD, in comparison to respective *ACE* genotypes in pre-menopause group. Taken together, FMD is related not only to menopause status, but also to *ACE* genotype. Interestingly, it has also been reported that the largest decline in FMD occurred between the pre-menopausal and perimenopausal (transitional) stages, not in the post-menopausal stage (Moreau, 2018).

### *ACE* D/I Polymorphism in Relation With FMD, ERFs, Age, and Menopause Status

The present study revealed that in pre-menopause group, plasma levels of NO and ET-1 were similar in women of *ACE* DD and *ACE* DI/II, but Ang II level was significantly higher in women of *ACE* DD than in women of *ACE* DI/II (see Table 5). Higher Ang II level has previously been observed in patients (both men and women with chronic heart failure) of *ACE* DD than *ACE* DI/II (Huang et al., 2004a,b). Recent studies have shown that Ang II promoted the onset and progression of vascular senescence, which was associated with vascular functional and structural changes, contributing to age-related vascular diseases (Min et al., 2009). Thus, the significantly higher Ang II level in women of *ACE* DD than in *ACE* DI/II is in support of the observation that FMD was prior in women of *ACE* DI/II to *ACE* DD in pre-menopausal group.

Further correlation analyses between FMD and ERFs revealed that in pre-menopause group, FMD in women of *ACE* DD was significantly affected by all the three ERFs, whereas FMD in women of *ACE* DI/II was only significantly affected by Ang II (see Table 6). Therefore, women of *ACE* DD had two extra ERFs: NO and ET-1 exerting significant effects on FMD. NO is the most important factor in the vasodilation system and plays an important role in age-related vascular dysfunction (Thijssen et al., 2016). ET-1 is the strongest vasoconstrictor, and an important regulator of vascular dysfunction during aging (Trindade et al., 2017). ET-1 has been known to act as a natural counterpart of vasodilator NO and contribute to vasocontraction through ETA and ETB receptors on smooth muscle. In contrast, activation of ETB receptors on endothelial cells results in transient Ca^2+^ and stimulation of endothelial nitric oxide synthase (eNOS), leading to vasodilation (Halcox et al., 2007; Tykocki et al., 2009). Physical factors such as shear stress, Ang II, and cytokines enhance secretion of ET-1, whereas NO, cyclic GMP, and prostacyclin reduce the release of ET-1 (Marasciulo et al., 2006; Houde et al., 2016). Under physiological conditions, the effects of ET-1 are precisely regulated through inhibitors or stimulators of ET-1. Furthermore, reduced function of ETB receptors or overactivated ETA receptors can eliminate the protective effect of NO on the vascular system (Bourque et al., 2011; Ohkita et al., 2012). Age-related impairment in vascular function has been proposed to be due to an imbalance between decrease in NO and increase in ET-1 (Godo et al., 2016; Stanhewicz et al., 2018). It is well-know that NO, ET-1 and Ang II are closely related and interact with each other to regulate the normal function of vascular endothelium (Godo et al., 2016: Somani et al., 2019). We speculate that the two extra ERFs: NO and ET-1 exerted negative effects on FMD, leading to the lower level of FMD in women of *ACE* DD in comparison to women of *ACE* DI/II in pre-menopause group. It is worth noticing that plasma ET-1 concentration is normally at nanogram level. Given that ET-1 has a molecular weight of 2491.9 g/mol, the statistically significant difference in ET-1 level in women of *ACE* DI/II between pre- and post-menopause groups (see Table 5) seems biologically meaningless. Thus, interpretation of the present data of plasma levels of ERFs in relation to FMD needs to be cautious.

The study revealed that in post-menopause group, none of the ERFs presented significant differences between women of *ACE* DD and *ACE* DI/II (see Table 5). Comparisons of ERFs levels in women of the same *ACE* genotype between pre- and post-menopause groups revealed that only ET-1 level in women of *ACE* DI/II was significantly higher in post-menopause group than in pre-menopause group (see Table 5). Estrogen has been reported to reduce aging rate of the vascular endothelium induced by Ang II (Imanishi et al., 2005), and to inhibit the release of ET-1 and reduce the response of blood vessels to Ang II (Lekontseva et al., 2010; Gohar et al., 2016). We speculate that women of *ACE* DI/II might be more sensitive to estrogen than DD, and estrogen reduction from pre-menopause to post-menopause led to reduced inhibition of estrogen on ET-1, resulting in the increased ET-1 level in post-menopausal women of *ACE* DI/II. ET-1 has been known to be an important regulator of vascular dysfunction during aging (Trindade et al., 2017). Its significant increase in post-menopausal women of *ACE* DI/II may be associated with the FMD reduction in women of *ACE* DI/II from pre-menopause stage to post-menopausal stage. The study revealed also that among all the ERFs, only ET-1 had significant correlation with FMD in post-menopausal women of *ACE* DD (see Table 6), which was believed to be associated with the significant reduction in percentage of *ACE* DD women at normal FMD level from pre-menopause to post-menopause (see Table 3).

## Conclusion

The present study observed that in the pre-menopause group, women of DI/II had higher FMD level than women of DD, and in the post-menopause group, FMD in women of DI/II was more age-dependent than in women of DD. The variations in FMD between women of different *ACE* genotypes were closely associated with plasma levels of ERFs, which varied with *ACE* D/I polymorphism and menopause status. In conclusion, women of *ACE* DI/II in pre-menopausal stage seem to have lower risk in developing cardiovascular disease than women of *ACE* DD, and in post-menopausal stage, the risk was similar in women of the two different *ACE* genotypes.

## Data Availability Statement

The raw data supporting the conclusions of this article will be made available by the authors, without undue reservation.

## Ethics Statement

The studies involving human participants were reviewed and approved by the Ethics Committee of Beijing Sport University (2019014H). The patients/participants provided their written informed consent to participate in this study.

## Author Contributions

LZ and J-GY designed the research study and analyzed the data. YL, WZ, and LY carried out the experiments. YL, J-GY, and WZ analyzed the results and performed statistical analysis in collaboration with LY. YL wrote the manuscript with help from J-GY and LZ. LZ and J-GY had primary responsibility for the final content. All authors read and approved the final manuscript.

## Conflict of Interest

The authors declare that the research was conducted in the absence of any commercial or financial relationships that could be construed as a potential conflict of interest.
